# The epidemiology of *Aedes*-borne arboviral diseases in Zhejiang, Southeast China: a 20 years population-based surveillance study

**DOI:** 10.3389/fpubh.2023.1270781

**Published:** 2023-10-23

**Authors:** Jiangping Ren, Zhiping Chen, Feng Ling, Ying Liu, Enfu Chen, Xuguang Shi, Song Guo, Rong Zhang, Zhen Wang, Jimin Sun

**Affiliations:** ^1^Zhejiang Provincial Center for Disease Control and Prevention, Hangzhou, China; ^2^Key Laboratory of Vaccine, Prevention and Control of Infectious Disease of Zhejiang Province, Hangzhou, China; ^3^Zhejiang Provincial Station of Emerging Infectious Disease Control and Prevention, Chinese Academy of Medical Sciences, Hangzhou, China

**Keywords:** dengue, chikungunya fever, zika, China, emergence

## Abstract

**Objective:**

*Aedes*-borne arboviral diseases were important public health problems in Zhejiang before the coronavirus disease 2019 (COVID-19) pandemic. This study was conducted to investigate the characteristics and change of the epidemiology of *Aedes*-borne arboviral diseases in the province.

**Methods:**

Descriptive analyses were conducted to summarize the epidemiology of *Aedes*-borne arboviral diseases during 2003–2022.

**Results:**

A total of 3,125 cases, including 1,968 indigenous cases, were reported during 2003–2022. Approximately three-quarters of imported cases were infected from Southeast Asia. The number of annual imported cases increased during 2013–2019 (*R*^2^ = 0.801, *p* = 0.004) and peaked in 2019. When compared with 2003–2012, all prefecture-level cities witnessed an increase in the annual mean incidence of imported cases in 2013–2019 (0.11–0.42 per 100,000 population vs. 0–0.05 per 100,000 population) but a drastic decrease during 2020–2022 (0–0.03 per 100,000 population). The change in geographical distribution was similar, with 33/91 counties during 2003–2012, 86/91 during 2013–2019, and 14/91 during 2020–2022. The annual mean incidence of indigenous cases in 2013–2019 was 7.79 times that in 2003–2012 (0.44 vs. 0.06 per 100,000 population). No indigenous cases were reported between 2020–2022. Geographical extension of indigenous cases was also noted before 2020—from two counties during 2003–2012 to 44 during 2013–2019.

**Conclusion:**

Dengue, chikungunya fever, zika disease, and yellow fever are not endemic in Zhejiang but will be important public health problems for the province in the post-COVID-19 era.

## 1. Introduction

Arboviral diseases are viral diseases transmitted by arthropods, predominantly mosquitoes, sandflies, and ticks. In the past five decades, there has been an unprecedented emergence of arboviral diseases, especially dengue, chikungunya, yellow fever, and zika, raising global concerns (1). Dengue, chikungunya, yellow fever, and zika are transmitted by *Aedes* mosquitoes and are similar in clinical symptoms, geographical and temporal distribution, prevention, and control strategy. These four viruses are single-stranded positive-sense RNA, of which dengue virus (DENV), yellow fever virus, and zika virus (ZIKV) belong to the genus *Flavivirus* in the family *Flaviviridae*, whereas chikungunya virus (CHIKV) is a member of the *Alphavirus* genus in the family *Togaviridae*. The clinical manifestations of these viruses are diverse, ranging from asymptomatic infection to mild and self-limited febrile illness, permanent severe disability, congenital anomalies, and early death, with no specific treatments that are currently available. Yellow fever can be prevented with vaccines, whereas the dengue vaccine is unsatisfactory, and no vaccine is currently available for chikungunya or zika.

Dengue is the most prevalent mosquito-borne disease and causes the heaviest health burden of any arbovirus. It was listed as one of 10 threats to global health in 2019 and a neglected tropical disease by the World Health Organization. Dengue is endemic in the tropics and subtropics, and now affects over half of the world’s population (2). It is estimated that there are 390 million dengue infections per year, of which 96 million manifest clinically (3). Geographically, Southeast Asia and South Asia are most devastatingly affected by dengue with the highest incidence of cases, deaths, and number of disability-adjusted life years, followed by Latin America and the Caribbean (3, 4).

Chikungunya was first identified in Tanzania in 1953 during a large outbreak of the disease. Traditionally, it was endemic in Africa and Asia, and in America since 2013 (5). Most infected people recover after some days, with some even having no clinical symptoms at all. However, some who are infected experience long-term impacts (mainly post chikungunya rheumatism), which can last for years (5). In one study, the prevalence of long-term disabilities for follow-up times of 6–12 months, 12–18 months, and > 18 months were 39.70%, 35.85%, and 28.20%, respectively (5). The disease burden from chronic CHIKV infections is significantly larger than that of acute infections (6).

Yellow fever is endemic in the tropical areas of Africa, and Central and South America (7). Through mass vaccination campaigns, the disease was successful controlled by the middle of the 20th century. However, it re-emerged in the endemic region during 2016–2018, leading to the first ever confirmed case of yellow fever in Asia in 2016 (8). The cause of the re-emergence was complicated, partly attributed to waning vaccine-derived or naturally acquired immunity, dwindling international vaccine supplies, and unsatisfactory vaccine coverage (9). It was estimated that, globally, 393.7–472.9 million people still require vaccination within at-risk districts to achieve the 80% population coverage threshold recommended by the World Health Organization. To protect at-risk populations, prevent international spread, and contain outbreaks rapidly, the Eliminate Yellow Fever Epidemics Strategy was launched by the World Health Organization in 2017 (7).

ZIKV was first isolated from rhesus macaque monkey in Uganda in 1947. Subsequently, sporadic human cases were reported in Africa and Asia. In the past decades, it gradually spread from Africa and Asia to Oceania and the Americas (10). At first, little attention was paid to zika due to its extremely low incidence and mild symptoms. However, since 2013, concerns about the disease have increased because of its dramatic increase in incidence and its association with the development of neurological diseases such as microcephaly and Guillain–Barré syndrome. The prevalence of microcephaly is approximately 3% in infants of mothers with confirmed or probable ZIKV infection during pregnancy (11), and that of ZIKV-associated Guillain–Barré syndrome is approximately 1.23% (12).

China also experienced the emergence and re-emergence of *Aedes*-borne arboviral diseases. Major dengue fever epidemics occurred in China in 2014 with 46,864 reported cases and in 2019 with 22,407 reported cases. It was estimated that the cost of dengue fever prevention and control in China in 2019 was approximately 3 billion Chinese Yuan (13). Outbreaks of indigenous chikungunya were reported in Guangzhou province in 2010, Zhejiang province in 2017, and Yunnan province in 2019. In February of 2016, the first zika case was confirmed in a Chinese traveler came from Venezuela; since then, imported cases have been identified sporadically. In the same year, yellow fever was first reported in China among 11 Chinese workers from Angola, also making the first confirmed case of yellow fever in Asia. Zhejiang province, located in the southeastern coastal area of China, has the most active economy, and the highest social mobility and population density, rendering it vulnerable to communicable diseases, especially travel-related imported disease. This study aimed to uncover the epidemiological characteristics of *Aedes*-borne arboviral diseases and their change in the past two decades in Zhejiang to provide guiding information for their control and prevention in the post-coronavirus disease 2019 (COVID-19) era.

## 2. Materials and methods

### 2.1. Data sources

Dengue cases were defined according to the Diagnostic Criteria and Principles of Management for Dengue (WS 216–2001, before 2008) (14) or Diagnostic Criteria for Dengue (WS 216–2008, after 2008) (15), or Diagnostic Criteria for Dengue (WS 216–2018, after August 2018) (16). Chikungunya fever was diagnosed according to the Diagnostic and Treatment Scheme for Chikungunya Fever (before August 2018) (17) or Diagnosis for Chikungunya Fever (WS/T 590–2018, after August 2018) (18). The Prevention and Control Scheme for ZIKV Disease (first edition, before April 2016) (19) or the Prevention and Control Scheme for ZIKV Disease (second edition, after April 2016) were used to confirm the cases of ZIKV disease (20). Yellow fever cases were defined with the Diagnosis and Treatment Scheme for Yellow Fever (before April 2016) (21) or the Prevention and Control Scheme for Yellow Fever (after April 2016) (22). All the data about *Aedes*-borne arboviral diseases in Zhejiang were collected from the Chinese National Notifiable Disease Surveillance System. Imported and indigenous cases were defined according to their epidemiological history. All data were provided anonymously without individual identifying information. Cases were recognized as imported if they were infected in places other than Zhejiang province; otherwise, they were recorded as indigenous. The annual demographic data of the counties in Zhejiang from 2004 to 2022 were collected from the Chinese National Notifiable Disease Surveillance System. A map of Zhejiang province was downloaded from National Earth System Science Data Sharing Infrastructure (23). The data were divided into three periods in this work: period one (2003–2012), period two (2013–2019), and period three (2020–2022).

### 2.2. Statistical analysis

The characteristics of the cases are presented as frequencies for categorical variables and median (inter-quartile range) or mean value ± standard deviations for quantitative variables. Continuous data were compared using the student’s *t*-test or analysis of variance. Categorical variables were analyzed with the chi-squared or Fisher’s exact tests. A significant difference was noted if *p* < 0.05. WPS Office 2016 (Kingsoft Software Service Co., Ltd., Beijing, China), SPSS software version 17.0 (SPSS Inc., Chicago, IL, United States), and R software (version 4.1.1) were used for all the descriptive and statistical analyses.

## 3. Results

### 3.1. General overview

**Figure 1:**
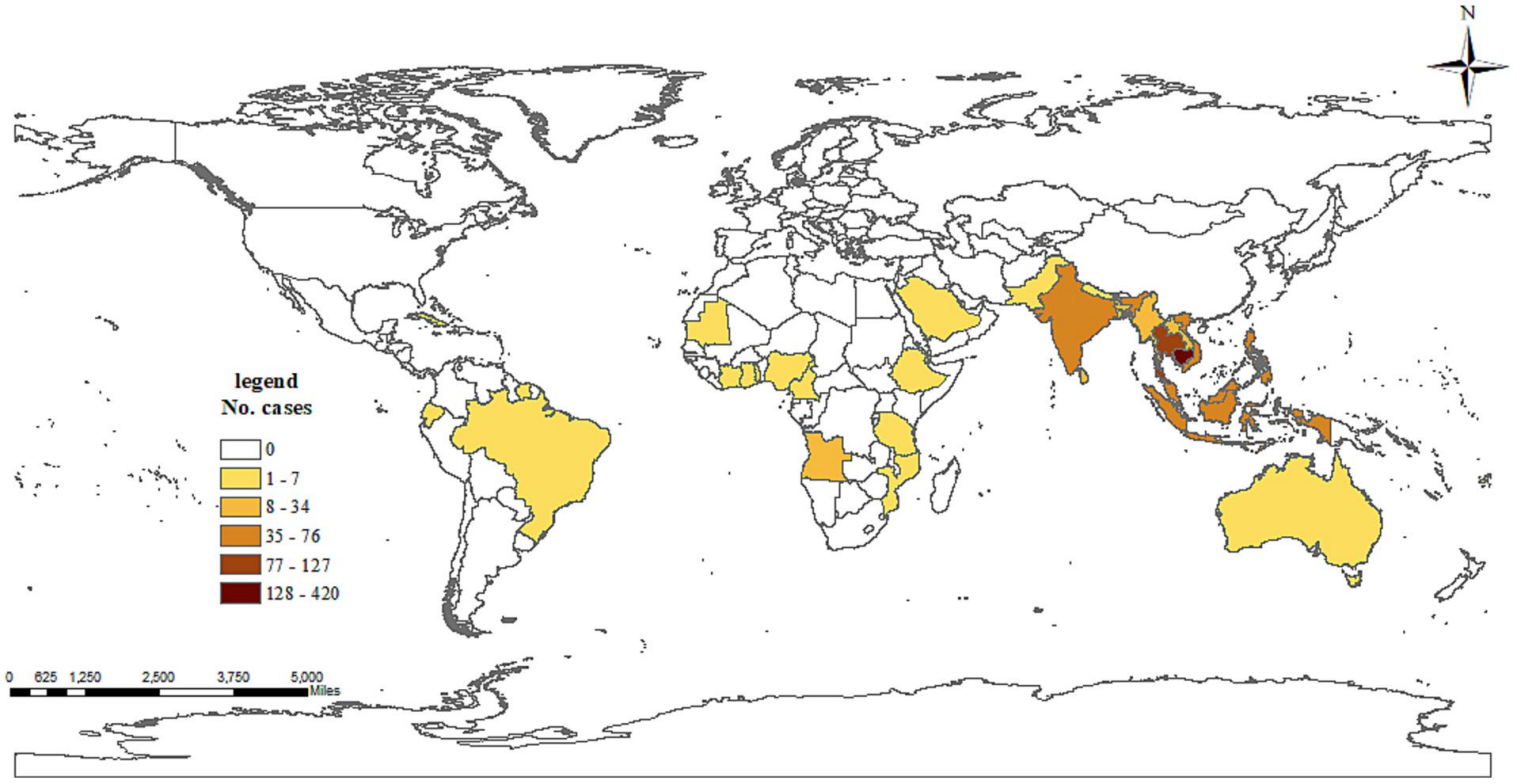
Number of dengue cases exported to Zhejiang, China, by country of origin during 2003–2022.

A total of 3,125 cases, with 3,124 symptomatic infections and one asymptomatic ZIKV infection, were reported during 2003–2022, with no deaths or severe cases reported. In those cases, 1,968 were indigenous, 1,081 were infected overseas, 75 were infected in other provinces in the Chinese mainland, and one imported case’s infection source was unidentified. Dengue was responsible for the overwhelming majority of *Aedes*-borne arboviral diseases in Zhejiang, with 1,965 indigenous and 1,125 imported cases, followed by chikungunya fever with three indigenous and 26 imported cases. No indigenous and six imported ZIKV infections were reported. No yellow fever was reported in Zhejiang during 2003–2022. Eight provinces in mainland China exported cases to Zhejiang, with 74 cases in 2013–2019 and one case in 2020. Yunnan and Guangzhou accounted for the majority of cases reported, as 41.33% and 40% of domestic imported cases were from those two provinces, respectively. According to the standard country or area codes for statistical use (M49), other than Europe, all five regions exported cases to Zhejiang, with Asia accounting for the vast majority (Table 1). At the sub-region level, cases from Southeast Asia ranked first in all three periods, and the proportion increased significantly over time (*Z* = 3.063, *p* = 0.002). Southern Asia was the second-most frequently reported infection source, but its proportion gradually decreased over time (*Z* = −3.152, *p* = 0.002). In total, 37 countries from the four regions exported cases to Zhejiang, and the five countries that exported the most cases were Cambodia (36.51% of imported cases), Thailand (11.51%), Vietnam (6.57%), the Philippines (6.14%), and India (5.71%), accounting for 71.05% of the overseas imported cases. The three countries that exported the most cases to Zhejiang were Cambodia (20% of imported cases), Bangladesh (8.57%), and Singapore (8.57%) during 2003–2012; Cambodia (38.01%), Thailand (11.85%), and Vietnam (6.40%) during 2013–2019; and the Philippines (21.74%), Cambodia (17.39%), and Singapore (17.39%) during 2020–2022. By year, for the imported cases from Cambodia, 80.33% (339/422) were reported in 2019. By disease, South-eastern Asia was the most common origin of overseas imported dengue and chikungunya in Zhejiang, accounting for 81.60% and 53.85% of cases, respectively (Figure 1). Southern Asia was also an important origin for chikungunya, as 38.46% of overseas imported cases were from this region. For zika, four out of six imported cases were from the Polynesian island nation of Samoa.

**Table 1:** The infection source of the imported *Aedes*-borne arboviral diseases in Zhejiang during 2003–2022.

Infection source		2003–2012 (*n*/%)	2013–2019 (*n*/%)	2020–2022 (*n*/%)	2003–2022 (*n*/%)
Asia	Southeast Asia	44 (62.86)	806 (75.82)	21 (91.30)	871 (75.35)
	South Asia	16 (22.86)	131 (12.32)	0 (0)	147 (12.72)
	West Asia	0 (0)	1 (0.09)	0 (0)	1 (0.087)
Africa	Sub-Saharan Africa	4 (5.71)	35 (3.29)	1 (4.35)	40 (3.46)
Americas	Latin America and the Caribbean	6 (8.57)	8 (0.75)	0 (0)	14 (1.21)
Oceania	Australia and New Zealand	0 (0)	1 (0.09)	0 (0)	1 (0.09)
	Melanesia	0 (0)	3 (0.28)	0 (0)	3 (0.26)
	Polynesia	0 (0)	4 (0.38)	0 (0)	4 (0.35)
Other provinces in China		0 (0)	74 (6.96)	1 (4.35)	75 (6.49)

### 3.2. Temporal distribution

**Figure 2:**
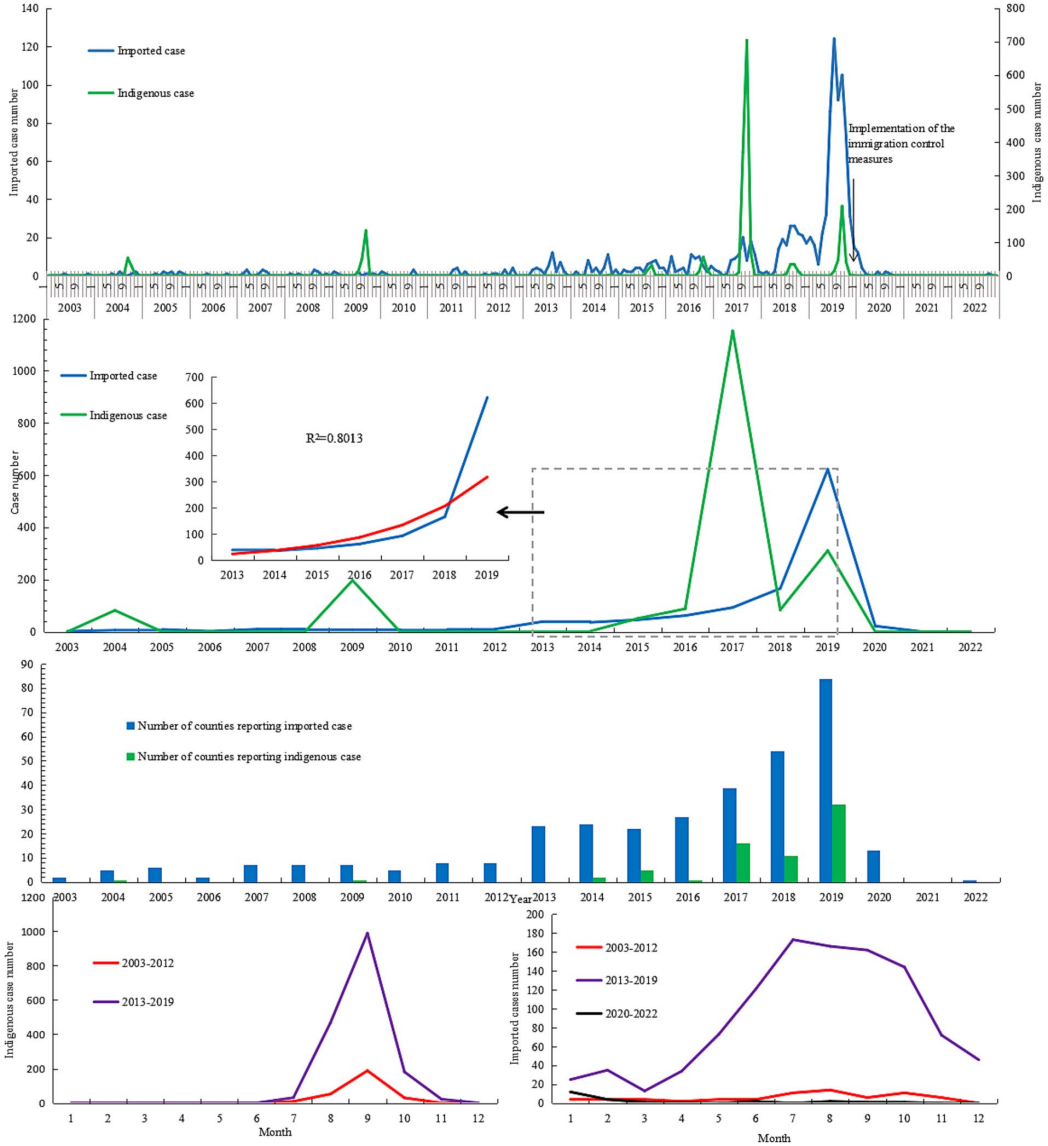
Temporal distribution of the *Aedes*-borne arboviral diseases in Zhejiang during 2003–2022.

Except in 2021, *Aedes*-borne infectious diseases were reported every year between 2003 and 2022 (Figure 2). The number of annual imported cases ranged from 2 to 10 during 2003–2012, with an annual mean incidence of 0.01 per 100,000 population. As a whole, the number of imported cases increased yearly during 2013–2019 [*R*^2^ = 0.801, log(*n*) = 2.744 + 0.431 (year-2012), *F* = 25.2, *p* = 0.004]. The annual imported case number exceeded 50 after 2016, and exceeded 100 after 2018 during this period. The annual mean incidence of imported cases during 2013–2019 was 0.27 per 100,000 population, 19.7 times that in 2003–2012. Since 2020, the annual imported case number drastically decreased due to the implementation of prevention and control measures to contain the spread of COVID-19. As a result, the annual mean incidence decreased by 95.67% and 14.85% in the period of 2020–2022 compared to that of 2013–2019 and 2003–2012, respectively. Approximately 70% of imported cases during 2020–2022 were reported between January and March 2020, before the implementation of the immigration control measures in China. Indigenous cases were reported in 2004, 2009, and 2014–2019; except for 2004, the annual case number in those years exceeded 50. The highest annual case number was recorded in 2017, with a total of 1,153 indigenous cases identified. The annual mean incidence of indigenous cases in 2013–2019 was 7.79 times that in 2003–2012 (0.44 vs. 0.06 per 100,000 population).

Imported cases were reported year-round, with a peak (70.70% of all notifications) between June and October (Figure 2). No significant difference in season distribution was noted between 2003–2012 and 2013–2019 (*χ*^2^ = 0.841, *p* = 0.840), in which the monthly peak was noted between July and October. During 2020–2022, more than half of the imported cases (52.17% of all notifications) were reported in January 2020. Indigenous cases were only reported in the months from July to November, and 59.76% cases were reported in September. The monthly distributions in 2003–2012 and 2013–2019 were significantly different (*χ*^2^ = 27.377, *p* < 0.001). No indigenous cases were identified in November during 2003–2012, the proportion of cases in September was higher than that in 2013–2019 (68.5% vs. 58.5%), and the proportion in October was lower (1.5% vs. 10.7%).

### 3.3. Spatial distribution

**Figure 3:**
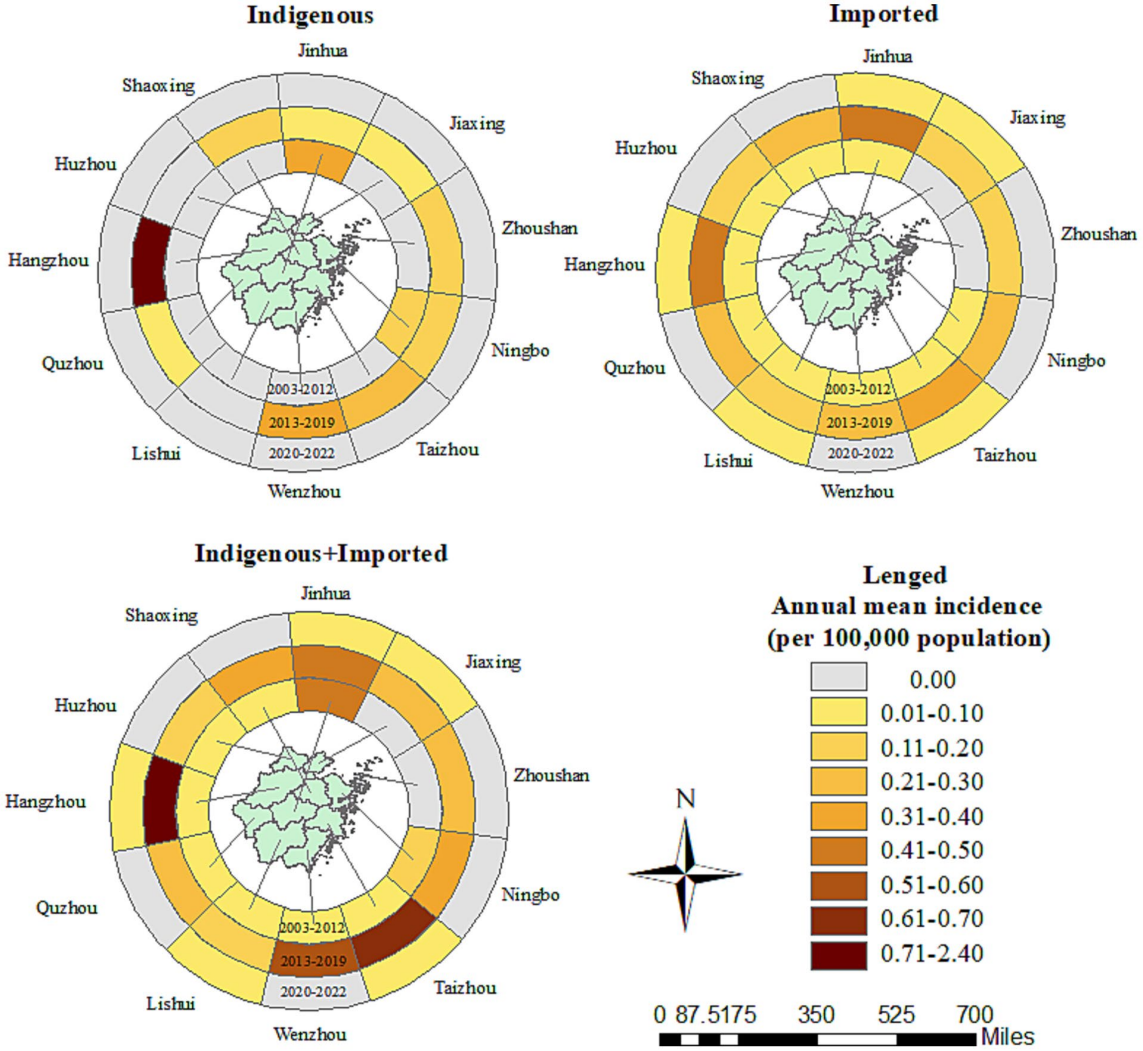
Prefecture-level city distribution of the *Aedes*-borne arboviral diseases in Zhejiang during 2003–2022.

All 11 prefecture-level cities reported *Aedes*-borne infectious diseases in Zhejiang between 2003 and 2022, with Hangzhou (0.86 per 100,000 population), Jinhua (0.34 per 100,000 population), and Taizhou (0.22 per 100,000 population) reporting the highest annual mean incidence. The three prefecture-level cities with the top annual mean incidence were Jinhua (0.41 per 100,000 population), Ningbo (0.24 per 100,000 population), and Lishui (0.16 per 100,000 population) during 2003–2012; Hangzhou (2.33 per 100,000 population), Taizhou (0.60 per 100,000 population), and Wenzhou (0.53 per 100,000 population) during 2013–2019; and Hangzhou (0.03 per 100,000 population), Taizhou (0.03 per 100,000 population), and Jiaxing (0.02 per 100,000 population) during 2020–2022 (Figure 3). Nine out of the 11 prefecture-level cities identified *Aedes*-borne infectious diseases between 2003 and 2012, with no cases reported in Jiaxing or Zhoushan. All 11 prefecture-level cities reported *Aedes*-borne infectious diseases during 2013–2019. In the period of 2020–2022, only five prefecture-level cities (Hangzhou, Taizhou, Jiaxing, Lishui, and Jinhua) reported imported cases, and no indigenous cases were identified.

**Figure 4:**
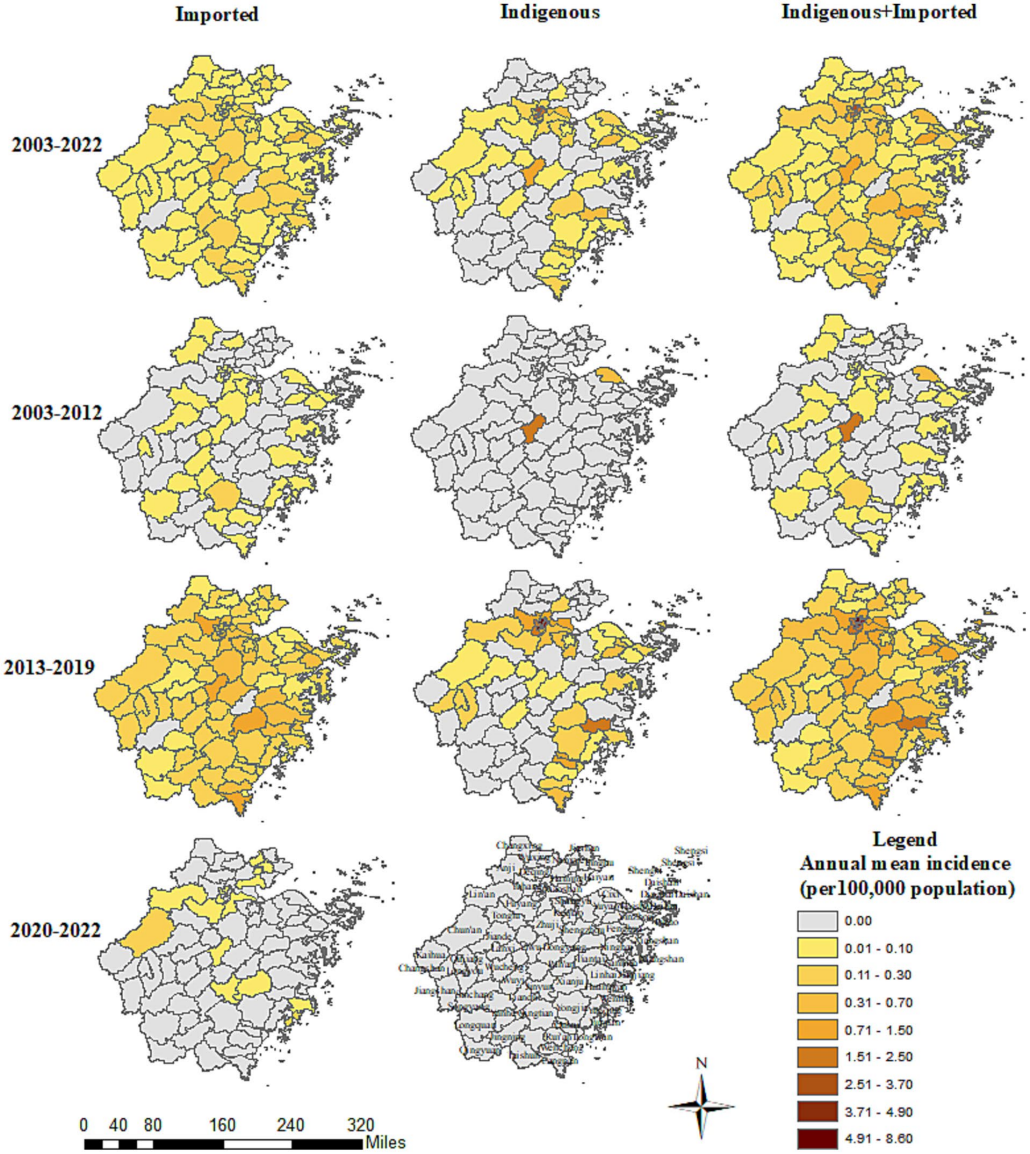
County distribution of the *Aedes*-borne arboviral diseases in Zhejiang during 2003–2022.

Imported cases of *Aedes*-borne infectious diseases were reported in all 11 prefecture-level cities in Zhejiang during 2003–2022. The three prefecture-level cities with the highest annual mean incidence of imported cases were Hangzhou (0.17 per 100,000 population), Jinhua (0.15 per 100,000 population), and Taizhou (0.13 per 100,000 population). The three prefecture-level cities with the highest annual mean incidence in the three study periods were notably different: Lishui (0.05 per 100,000 population), Ningbo (0.02 per 100,000 population), and Huzhou (0.02 per 100,000 population) during 2003–2012; Hangzhou (0.42 per 100,000 population), Jinhua (0.41 per 100,000 population), and Taizhou (0.34 per 100,000 population) during 2013–2019; and Hangzhou (0.03 per 100,000 population), Taizhou (0.03 per 100,000 population), and Jiaxing (0.02 per 100,000 population) during 2020–2022. All the prefecture-level cities witnessed an increase in the annual mean incidence of imported cases in 2013–2019 compared to 2003–2012, which was most notable in Hangzhou (0.42 vs. 0.02 per 100,000 population). In contrast, in 2020–2022, all the prefecture-level cities had a drastic decrease in the annual mean incidence of imported cases. The number of prefecture-level cities that reported imported cases was nine during 2003–2012, 11 during 2013–2019, and five during 2020–2022. The proportion of counties that reported imported cases was 33/91 during 2003–2012, 86/91 during 2013–2019, and 14/91 during 2020–2022. No imported cases were reported in Dongtou, Pan’an, Shengsi, Suichang, or Xihufengjingmingsheng across the whole study period (Figure 4). The top-five counties with the highest annual mean incidence of imported cases were totally different during the different periods: Qingtian (0.18 per 100,000 population), Beilun (0.10 per 100,000 population), Haishu (0.08 per 100,000 population), Liandu (0.08 per 100,000 population), and Wencheng (0.07 per 100,000 population) during 2003–2012; Yiwu (1.05 per 100,000 population), Binjiang (0.85 per 100,000 population), Xianju (0.78 per 100,000 population), Yuhang (0.72 per 100,000 population), and Cangnan (0.71 per 100,000 population) during 2013–2019; and Yuhuan (0.10 per 100,000 population), Chunan (0.10 per 100,000 population), Xihu (0.09 per 100,000 population), Jinyun (0.08 per 100,000 population) and Fuyang (0.08 per 100,000 population) during 2020–2022.

Indigenous cases were recorded in 9/11 prefecture-level cities during 2003–2022. Hangzhou (0.69 per 100,000 population) reported the highest annual mean incidence of indigenous cases, followed by Jinhua (0.19 per 100,000 population) and Wenzhou (0.11 per 100,000 population). No indigenous cases were reported in Huzhou or Lishui. In the period of 2003–2012, indigenous cases were only reported in two counties: Cixi in Ningbo and Yiwu in Jinhua. The annual mean incidence during 2003–2012 was 0.13 and 0.40 per 100,000 population for these two prefecture-level cities, and 0.61 and 1.99 per 100,000 population for these two counties, respectively (Figure 4). During 2013–2019, 9/11 prefecture-level cities and 44/91 counties confirmed indigenous cases. The three cities with the highest annual mean incidence of indigenous cases during 2013–2019 were Hangzhou (1.91 per 100,000 population), Wenzhou (0.31 per 100,000 population), and Taizhou (0.26 per 100,000 population). The five counties with the highest annual mean incidence were Gongshu (8.04 per 100,000 population), Xihufengjingmingsheng (5.60 per 100,000 population), Shangcheng (4.29 per 100,000 population), Huangyan (1.95 per 100,000 population), and Xihu (1.84 per 100,000 population). Other than Jinhua, Huzhou, and Lishui, all the prefecture-level cities had a higher annual mean incidence of indigenous cases in the period of 2013–2019.

### 3.4. Demographic characteristics

There were 1,716 male and 1,409 female cases during 2003–2022, with a male:female gender ratio of 1.218:1. Males outnumbered females for imported cases, but the situation was reversed for indigenous cases. Compared with indigenous cases, there were significantly more male than female imported cases in both 2003–2012 and 2013–2019 (*p* < 0.001). No significant differences in the gender distributions for imported cases were noted between the three periods (*χ*^2^ = 0.150, *p* = 0.928). For indigenous cases, the male proportion in 2013–2019 was significantly higher than that in 2003–2012 (*χ*^2^ = 14.441, *p* < 0.001).

**Figure 5:**
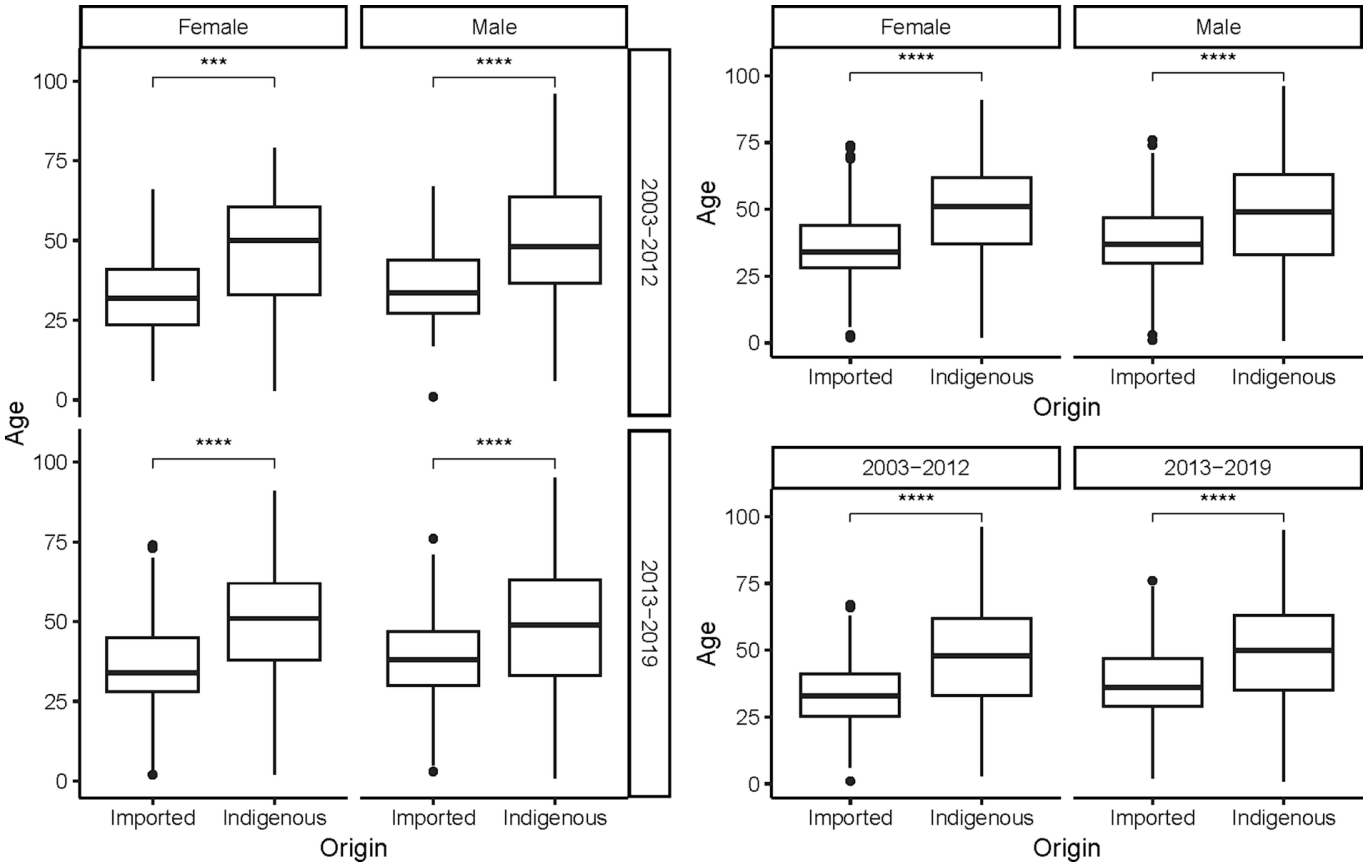
Age distribution of the *Aedes*-borne arboviral diseases with different infectious origins by different genders and periods. Ns *p* > 0.05, ^*^p ≤ 0.05, ^**^p ≤ 0.01, ^***^p ≤ 0.001, and ^****^p ≤ 0.0001.

**Figure 6:**
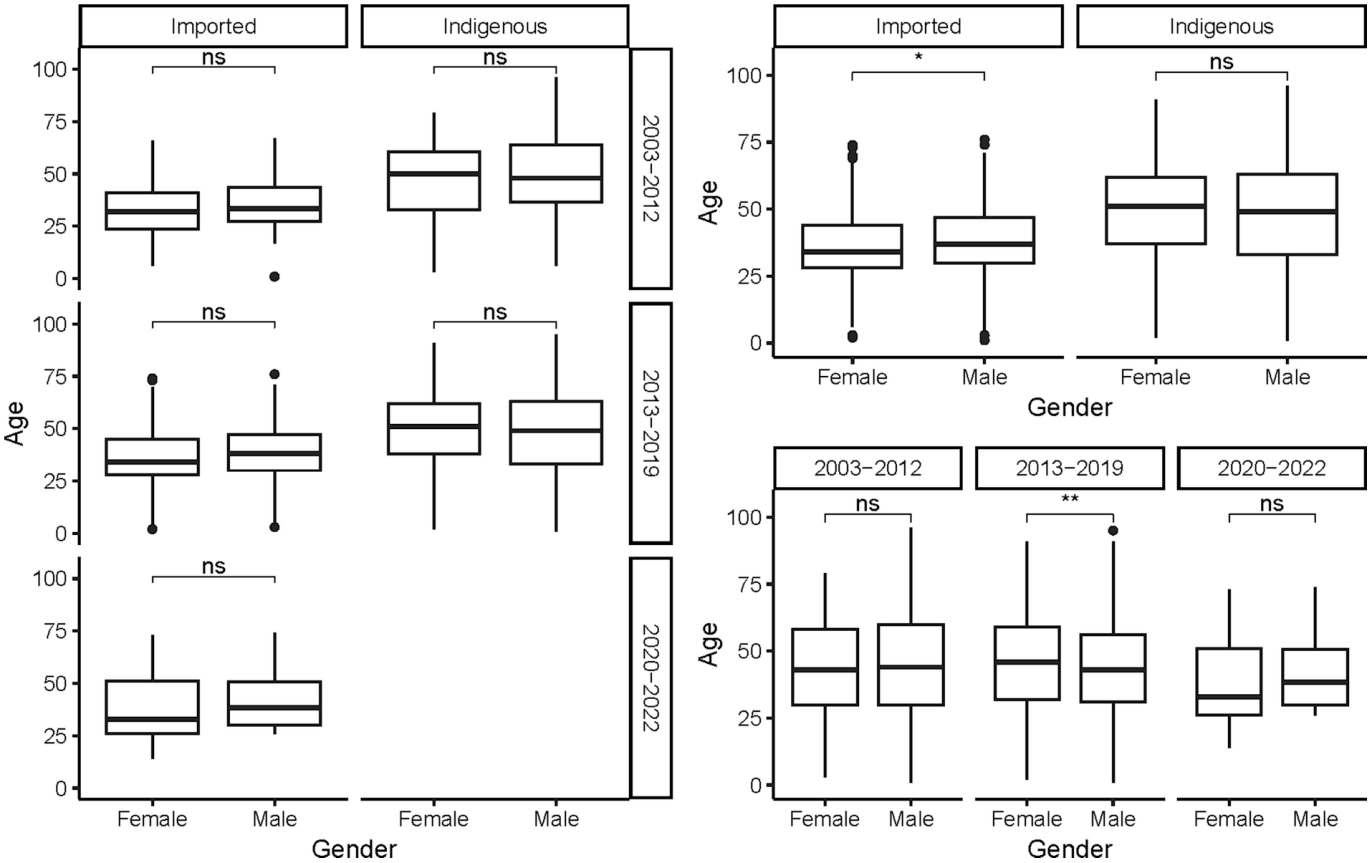
Age distribution of the *Aedes*-borne arboviral diseases of different genders by different infectious origins and periods. Ns *p* > 0.05, ^*^p ≤ 0.05, ^**^p ≤ 0.01, ^***^p ≤ 0.001, and ^****^p ≤ 0.0001.

**Figure 7:**
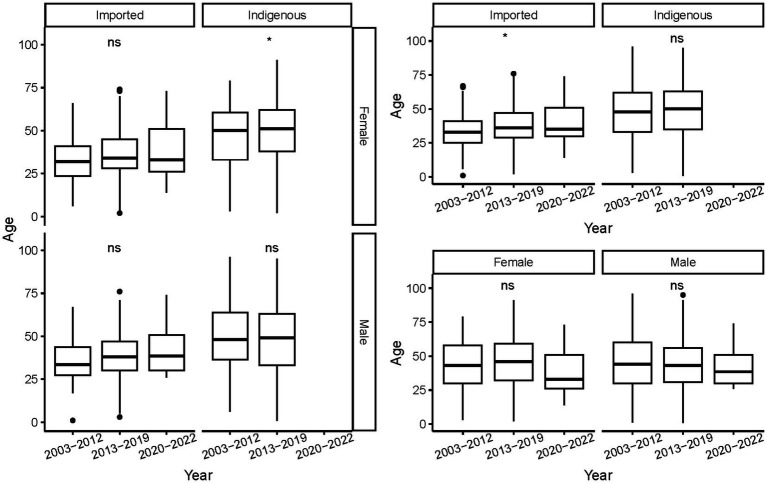
Age distribution of the *Aedes*-borne arboviral diseases of different periods by different genders and infectious origins. Ns *p* > 0.05, ^*^p ≤ 0.05, ^**^p ≤ 0.01, ^***^p ≤ 0.001, and ^****^p ≤ 0.0001.

The ages of the cases ranged from 9 months to 96 years, with a mean of 44.78 ± 17.103 years. On average, indigenous cases were older than the imported ones, regardless of gender and period (*p* < 0.001, Figure 5). As a whole, female cases were significantly older than male cases in 2013–2019 (*t* = −3.271, *p* = 0.0011), whereas male imported cases were significantly older than female imported cases during 2003–2002 (*t* = 2.040, *p* = 0.042). No significant difference in age was noted for cases of different genders from different periods and infectious origins (*p* > 0.05, Figure 6). For the female indigenous cases, those from 2013–2019 were significantly older than those from 2003–2012 (*t* = −2.283, *p* = 0.023, Figure 7). For the imported cases from different periods, a significant difference in age distribution was identified (*F* = 3.188, *p* = 0.042), whereas no difference was noted when subdivided into different genders (*p* > 0.05).

**Figure 8:**
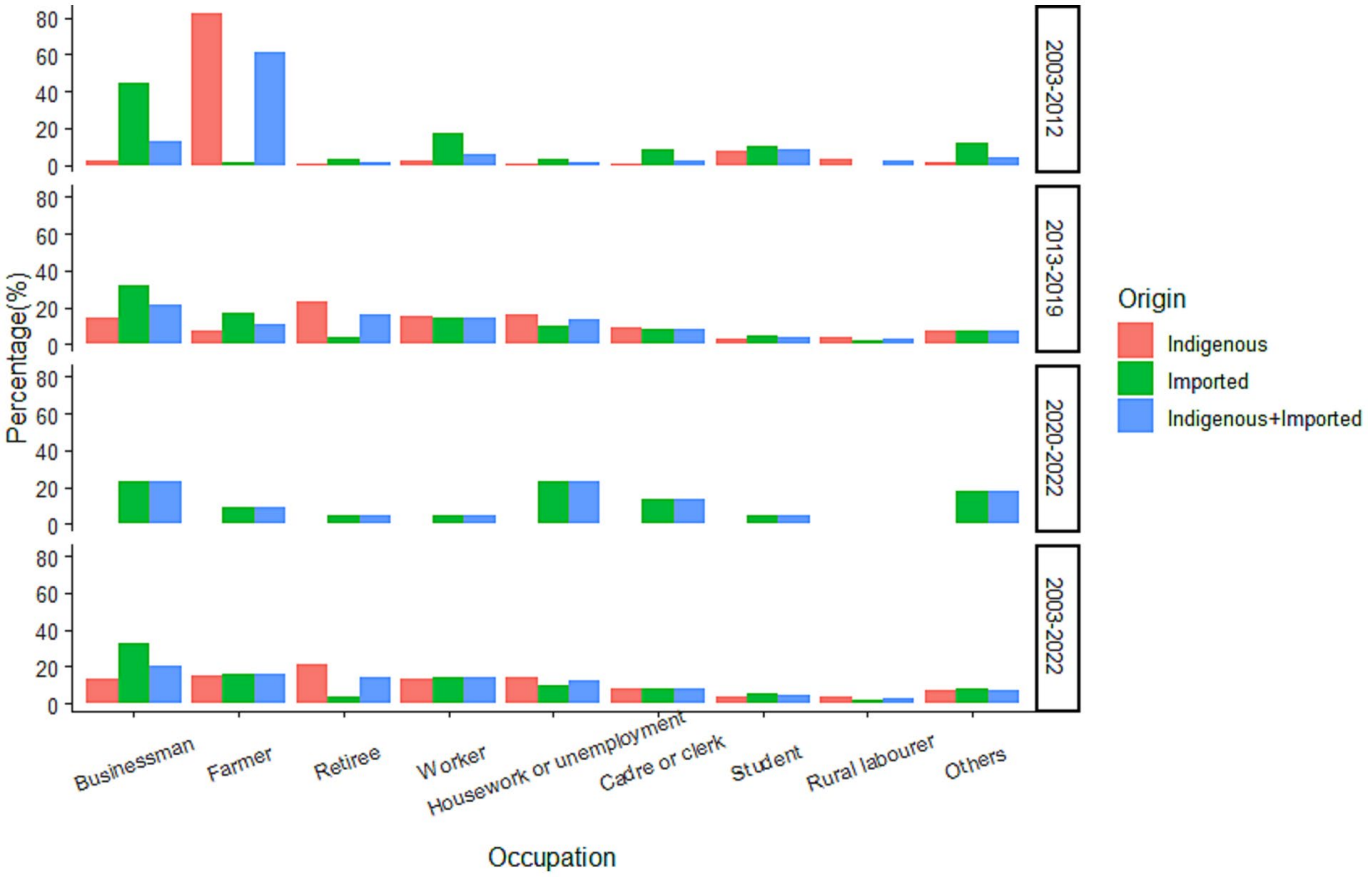
Occupational distribution of the *Aedes*-borne arboviral diseases in different periods.

Occupational information was available for 2,983 cases, with businessperson (20.52%), farmer (15.59%), retiree (14.58%), worker (13.95%), and housework or unemployment (12.54%) as the five most frequently mentioned occupations (Figure 8). Overall, the occupation distribution was different for cases of different infectious origins (*χ*^2^ = 304.128, *p* < 0.0001), with indigenous cases mainly reporting occupations of retiree (21.17%), farmer (15.17%), housework or unemployment (14.20%), worker (13.67%), and businessperson (13.18%); and imported cases reporting occupations of businessperson (32.77%), farmer (6.29%), worker (14.41%), and housework or unemployment (9.76%). For the cases from 2003–2012 and 2013–2019, the occupation distribution was also significantly different (*χ*^2^ = 503.889, *p* < 0.001). The top-four occupations in 2003–2012 were farmer (61.28%), businessperson (13.16%), student (8.27%), and worker (6.02%), whereas the five most frequently reported occupations in 2013–2019 were businessperson (21.22%), retiree (15.99%), worker (14.81%), housework or unemployment (13.58%), and farmer (11.13%). Significant differences were also noted when cases were further subdivided into different infectious origins (*p* < 0.05). For the indigenous cases, in 2003–2012, the occupations of most reports were mainly farmer (82.23%) and student (7.61%), whereas retiree (23.61%), housework or unemployment (15.82%), worker (15.04%), and businessperson (14.50%) were the four most frequently reported occupations in 2013–2019. For the imported cases, the most common occupations were businessperson (44.93%) and worker (17.39%) for cases during 2003–2012; whereas businessperson (32.16%), farmer (17.45%), and worker (14.42%) were the most common in 2013–2019.

## 4. Discussion

The past decades have seen a global increase in the frequency, magnitude, and geographical expansion of *Aedes*-borne arboviral diseases. The cause of their emergence and re-emergence is complicated and includes many aspects, such as climate change, globalization, virus evolution, urbanization, insufficient mosquito control, and virus and vector adoption. The primary vector for *Aedes*-borne arboviral diseases is *Aedes aegypti*, which is concentrated in the tropical and subtropical parts of the world and has demonstrated suitability foci in 188 countries/territories (24). *Aedes albopictus* is the second-most important vector for DENV, ZIKV, and CHIKV. Its range extends from the tropics into the temperate parts of the world, with suitability foci in 197 countries/territories. In mainland China, the geographical distribution of *A. aegypti* is limited, as it is only found near the border or in coastal areas of Yunnan, Guangxi, Guangdong, and Hainan provinces (25). Vector surveillance has indicated that the geographic distribution of *A. aegypti* expanded in Yunnan province but contracted in Guangxi, Guangdong, and Hainan provinces in recent years (25). *A. albopictus* has been found throughout tropical, subtropical, and temperate zones in China, spanning most of the area from Hainan province to Liaoning province, and is the dominant mosquito species in residential areas. In Zhejiang province, *A. albopictus* is the primary vector of *Aedes*-borne arboviral diseases, and *A. aegypti* is not found. A study conducted in mainland China indicated that *A. aegypti* has its own unique ecological niches, and the influence factors for its spatial distribution include annual mean temperature, isothermality, temperature seasonality, rural residential land, and rivers (26).

Globally, dengue is the most prevalent and widely distributed *Aedes*-borne arboviral disease, as 111 countries/territories had reported the autochthonous transmission of DENV between 1952 and 2017 (24). In this period, the overall numbers of countries/territories reporting autochthonous occurrences of CHIKV, ZIKV, and yellow fever virus was 106, 85, and 43, respectively (24). Other than malaria, dengue accounted for the overwhelming majority of imported infectious disease in mainland China (27), and the ratio of indigenous to imported cases was approximately 6.43:1 (25). Likewise, other than malaria, dengue was the most imported infectious disease in Zhejiang (28), but the ratio of indigenous to imported cases (1.75:1) was significantly lower than that in mainland China (25). The remarkably low indigenous-to-imported case ratio was attributed to the advantages of early diagnose, social mobilization, health education, vector control, and quick emergency response that characterize disease control and prevention in Zhejiang (29). More than four-fifths of overseas imported cases in the province were infected in Southeast Asia. Globally, Southeast Asia was also a major source of imported dengue (30). Thailand, Myanmar, Indonesia, and the Philippines were the top-four countries from which dengue was imported, whereas the four countries that exported the most dengue cases to Zhejiang were Cambodia, Thailand, Vietnam, and the Philippines. A total of 337 cases were infected in Cambodia in 2019, accounting for 59.86% and 32.13% of overseas imported dengue cases in 2019 and 2003–2022, respectively. Cambodia was also the most common origin of overseas imported dengue cases in mainland China in 2019, accounting for 55.9% of cases (31). To improve and strengthen cooperation in culture and tourism, Cambodia and China designated 2019 as the “China–Cambodia culture and tourism year,” and a variety of activities were jointly organized to celebrate the year. There were 19 airlines operating some 500 direct flights per week between the two nations that year. Thus, in the first 10 months of 2019, Chinese tourists topped the list of foreign visitors coming to Cambodia at 2.02 million—a 24.4% year-on-year increase, accounting for 38% of all of Cambodia’s international tourists. In same year, Cambodia had endured the most serious dengue outbreak in the past few years (32). All of these factors led to a significant increase in the number of imported dengue cases in China and specifically Zhejiang from Cambodia.

Although chikungunya was the second-most frequently reported *Aedes*-borne arboviral disease in Zhejiang, the number of cases was obviously lower than that of dengue. All three indigenous cases were reported in Quzhou in 2017, representing the second autochthonous CHIKV transmission in mainland China (33). The indigenous-to-imported case ratio in Zhejiang was also significantly lower than that of mainland China (0.12:1 vs. 4.52:1) but similar to that of Taiwan (33, 34). Southeast and South Asia were the largest sources of chikungunya in Zhejiang; Thailand, Bangladesh, and Myanmar were the countries that exported the most cases, similar to the situation for the whole nation (33). Southeast and South Asia were also a major source of chikungunya in Japan, but the top-three countries were Indonesia, India, and the Philippines (35). The imported zika cases in Zhejiang were mainly reported from a tour group traveling to Fiji and Samoa, whereas approximately two-thirds of the imported zika cases in mainland China were from Venezuela (36). No indigenous zika cases were reported in mainland China until now. However, in a retrospective study, ZIKV was isolated from a local man with a fever of unknown origin residing in Ruili, a China-Myanmar border city, Yunnan province, Southwest China, who did not travel overseas (37). In another study conducted in Guangxi province, Southwest China, healthy individuals with no overseas experience and negative for DENV and West Nile virus were found to be serologically positive for ZIKV and had micro-neutralization antibodies (38). Pigs, chickens, and sheep were also found to be seropositive for ZIKV in Guizhou province, Southwest China (39). Besides, strains of ZIKV were isolated from wild *Anopheles sinensis*, *Culex tritaeniorhynchus*, *Culex quinquefasciatus,* and *Armigeres subalbatus* in Southern China (40–42). A study of vector competence for ZIKV in China indicated that *A. aegypti* had the highest transmissibility, followed by *A. albopictus*, whereas *C. quinquefasciatus* had no transmission ability (43). Another study conducted in China indicated that *A. subalbatus* was a potential vector for ZIKV (44). All the above-mentioned studies suggested that there might be restricted autochthonous ZIKV transmission in Southern China, but further research was needed.

Coupled with the above-mentioned global emergence and re-emergence, Zhejiang witnessed an increase in the frequency, magnitude, and geographical distribution of *Aedes*-borne arboviral diseases, especial for dengue, only a few years before the COVID-19 pandemic. In the 3 years after the identification of severe acute respiratory syndrome coronavirus 2 (SARS-CoV-2), no indigenous cases of *Aedes*-borne arboviral diseases were reported in Zhejiang, and the number of imported cases was drastically decreased, especially after the execution of immigration control measures to control and prevent the import of SARS-CoV-2. The results proved that Zhejiang is not an endemic province for dengue, zika, chikungunya, or yellow fever, and that the identified autochthonous transmissions were due to imported infected. Travelers played a key role in the introduction of viruses for *Aedes*-borne arboviral diseases in non-endemic areas. It was confirmed that passenger flows *via* airline travel from countries experiencing *Aedes*-borne arboviral diseases epidemics were positively correlated to the number of imported cases in China, Korea, and the United States (45–47). A 10% increase in the volume of air travelers from dengue-endemic countries was associated with a 5.9% increase in detected cases of imported dengue in China, and a 10% increase from chikungunya-endemic countries was associated with a 5.2% increase in imported chikungunya in the United States. A study in two dengue-high-risk areas of China indicated that one of the most important influence factors for dengue fever occurrence was the number of imported cases (48). Non-pharmaceutical interventions (NPIs) to mitigate the transmission of SARS-CoV-2 had different effects on vector-borne communicable diseases in different regions. For endemic diseases, the imposition of NPIs was related to increased of case numbers, such as tick-borne encephalitis in Germany, Ross River virus in Australia, and dengue fever in Peru (49–51). In contrast, for non-endemic vector-borne communicable diseases, NPIs were associated with a decline in case number, such as dengue and malaria cases in Australia and Germany, and dengue in China (49, 50, 52). The drop in the international passenger flight was believed to be the main reason for the decline of vector-bore communicable disease in non-endemic regions.

The determinants for the occurrence of vector-borne disease are complicated and numerous, including the presence and abundance of vectors, ecoclimatic conditions, the density of the human population, access of vectors to humans, and the underlying disease immunity of the population (53). As a non-endemic province, the introduction of the virus was the primary determinant for its transmission in Zhejiang. Regions with a higher frequency of overseas exchange and cooperation, larger population mobility, and denser population were at greater risk of *Aedes*-borne arboviral diseases in Zhejiang. Males who were 20–50 years of age, more physically active, and were more likely to travel overseas dominated the imported cases of the province, similar to the situation in mainland China and Korea (26, 47). Compared with imported cases, indigenous cases were older and more likely to be female on average, whereas in mainland China indigenous cases were younger than those in Zhejiang (54). The distribution of occupation, both for imported and indigenous cases, was similar between mainland China and Zhejiang province (54).

## 5. Conclusion

Dengue, chikungunya, zika and yellow fever were not endemic in Zhejiang province, and Southeast Asia was the major source of the imported cases. Before the COVID-19 pandemic, Zhejiang experienced a significant increase in the case number and an extension of the geographical distribution of *Aedes*-borne arboviral diseases, including imported and indigenous cases. Following the implementation of the NPIs to mitigate the transmission of SARS-CoV-2, only a few imported cases were reported during 2020–2022, and no indigenous cases were confirmed. In the post-COVID-19 era, with the recovery in international population mobility and global trade, there will be a worldwide emergence and re-emergence of *Aedes*-borne arboviral diseases, and Zhejiang will witness a fast rise in case number, both imported and indigenous, and an extension in the geographical distribution of the diseases. Therefore, intensive surveillance, professional training, health education, vector control, and social mobilization are highly needed.

## Data availability statement

The raw data supporting the conclusions of this article will be made available by the authors, without undue reservation.

## Ethics statement

The studies involving humans were approved by the Ethics Committee of the Zhejiang Provincial Center for Disease Control and Prevention (No. 2020-021). The studies were conducted in accordance with the local legislation and institutional requirements. The ethics committee/institutional review board waived the requirement of written informed consent for participation from the participants or the participants’ legal guardians/next of kin because as an important part of ongoing public health surveillance, the collection and analysis of data on *Aedes*-borne arboviral diseases by public health workers in charge of risk assessment and policy proposals are exempt from written informed consent. It is authorized by the Law of the People’s Republic of China on the Prevention and Treatment of Infectious Diseases. All of the collected data were supplied anonymously and no individual identifying information was provided.

## Author contributions

JR: Data curation, Formal analysis, Investigation, Methodology, Writing – original draft, Writing – review & editing. ZC: Data curation, Supervision, Writing – original draft. FL: Data curation, Supervision, Writing – original draft. YL: Data curation, Investigation, Writing – original draft. EC: Data curation, Investigation, Writing – original draft. XS: Investigation, Writing – original draft. SG: Investigation, Writing – original draft. RZ: Investigation, Writing – original draft. ZW: Conceptualization, Resources, Supervision, Writing – original draft. JS: Conceptualization, Resources, Supervision, Writing – original draft.
